# The Nuclear Orphan Receptor NR2F6 Promotes Hepatic Steatosis through Upregulation of Fatty Acid Transporter CD36

**DOI:** 10.1002/advs.202002273

**Published:** 2020-09-21

**Authors:** Bing Zhou, Lijing Jia, Zhijian Zhang, Liping Xiang, Youwen Yuan, Peilin Zheng, Bin Liu, Xingxing Ren, Hua Bian, Liwei Xie, Yao Li, Jieli Lu, Huijie Zhang, Yan Lu

**Affiliations:** ^1^ The Key Laboratory of Metabolism and Molecular Medicine of the Ministry of Education Department of Endocrinology and Metabolism Fudan Institute for Metabolic Diseases Zhongshan Hospital Fudan University Shanghai 230032 P. R. China; ^2^ Department of Endocrinology Shenzhen People's Hospital The Second Clinical Medical College, Jinan University, The First Affiliated Hospital of Southern University of Science and Technology Shenzhen Guangdong 518020 P. R. China; ^3^ Department of Endocrinology and Metabolism Shanghai General Hospital Shanghai Jiao Tong University School of Medicine Shanghai 201620 P. R. China; ^4^ Department of Endocrinology and Metabolism Nanfang Hospital Southern Medical University Guangzhou Guangdong 510515 P. R. China; ^5^ State Key Laboratory of Applied Microbiology Southern China Guangdong Provincial Key Laboratory of Microbial Culture Collection and Application Guangdong Open Laboratory of Applied Microbiology Guangdong Institute of Microbiology Guangdong Academy of Sciences Guangzhou Guangdong 510070 P. R. China; ^6^ Department of Laboratory Animal Science Shanghai Jiao Tong University School of Medicine Shanghai 200025 P. R. China; ^7^ Department of Endocrinology and Metabolism Ruijin Hospital Shanghai Jiao Tong University School of Medicine Shanghai 200025 P. R. China

**Keywords:** CD36, non‐alcoholic fatty liver disease, NR2F6, nuclear receptors, obesity

## Abstract

Nuclear receptors (NRs) are a superfamily of transcription factors which sense hormonal signals or nutrients to regulate various biological events, including development, reproduction, and metabolism. Here, this study identifies nuclear receptor subfamily 2, group F, member 6 (NR2F6), as an important regulator of hepatic triglyceride (TG) homeostasis and causal factor in the development of non‐alcoholic fatty liver disease (NAFLD). Adeno‐associated virus (AAV)‐mediated overexpression of NR2F6 in the liver promotes TG accumulation in lean mice, while hepatic‐specific suppression of NR2F6 improves obesity‐associated hepatosteatosis, insulin resistance, and methionine and choline‐deficient (MCD) diet‐induced non‐alcoholic steatohepatitis (NASH). Mechanistically, the fatty acid translocase CD36 is identified as a transcriptional target of NR2F6 to mediate its steatotic role. NR2F6 is able to bind directly onto the CD36 promoter region in hepatocytes and increases the enrichment of nuclear receptor coactivator 1 (SRC‐1) and histone acetylation at its promoter. Of pathophysiological significance, NR2F6 is significantly upregulated in the livers of obese mice and NAFLD patients. Moreover, treatment with metformin decreases NR2F6 expression in obese mice, resulting in suppression of CD36 and reduced hepatic TG contents. Therefore, these results provide evidence for an unpredicted role of NR2F6 that contributes to liver steatosis and suggest that NR2F6 antagonists may present a therapeutic strategy for reversing or treating NAFLD/NASH pathogenesis.

## Graphical abstract

NR2F6 (nuclear receptor subfamily 2, group F, member 6) is increased in the livers of obese mice and patients with non‐alcoholic fatty liver disease. It transcriptionally upregulates the fatty acid transporter CD36 to enhance fatty acid uptake in hepatocytes, thereby promoting liver steatosis. These findings identify an important role for NR2F6 in the regulation of hepatic lipid homeostasis.

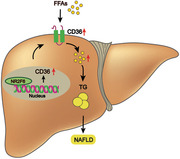

## 1 Introduction

Due to the high prevalence of obesity, non‐alcoholic fatty liver disease (NAFLD), characterized by excessive triglycerides (TGs) in hepatocytes, has become the most common liver disease worldwide.^[^ ^1^, ^2^ ^]^ NAFLD can further progress to develop nonalcoholic steatohepatitis (NASH), which is accompanied by persistent liver injury, inflammation, and varying degree of fibrosis.^[^ ^1^, ^2^ ^]^ Moreover, patients with NAFLD or NASH have increased risk for severe metabolic diseases, including type 2 diabetes mellitus (T2DM), dyslipidemia, and hypertension.^[^ ^1^, ^2^ ^]^ Although many pathophysiological factors have been implicated in the initiation and progression of NAFLD, such as insulin resistance,^[^ ^3^ ^]^ fructose consumption,^[^ ^4^ ^]^ dysregulation of hepatokines,^[^ ^5^ ^]^ and changes of gut microbiota,^[^ ^6^ ^]^ the molecular basis that promote the accumulation of TGs in the hepatocytes remains to be elucidated.

TGs are synthesized from fatty acids (FFAs), which are derived from lipolysis in adipocytes and de novo lipogenesis (DNL) in hepatocytes.^[^ ^7^ ^]^ At the molecular level, enzymes involved in the process of DNL, including Fasn, Acaca, and Scd‐1, is regulated by two transcription factors in response to insulin and glucose: sterol regulatory element binding protein 1c (SREBP‐1c) and carbohydrate‐responsive element binding protein (ChREBP), respectively.^[^ ^7^ ^]^ On the other hand, circulating FFAs are transported into hepatocytes by fatty acid transporters, including fatty acid translocase (FAT/CD36), plasma membrane‐associated fatty acid‐binding protein (FABPpm), and a family of fatty acid transport proteins (FATP1‐6).^[^ ^7^ ^]^ Among these transporters, CD36 is a type B scavenger receptor which has been studied extensively for its important role in the pathogenesis of NAFLD and T2DM. For example, overexpression of CD36 enhanced fatty acid uptake and TG accumulation in the livers of healthy mice and human hepatocytes.^[^ ^8^, ^9^ ^]^ Besides, human studies have shown that the rates of FFAs uptake in liver were dramatically increased in obese subjects due to elevated expression of CD36.^[^ ^7^, ^10^ ^]^ Thus, hepatic CD36 expression is closely associated with liver steatosis.

The nuclear receptor (NR) superfamily is comprised of 49 members in mammalians and coordinates many aspects of biological functions, including development, reproduction, and metabolism.^[^ ^11^ ^]^ Decades of studies have well demonstrated that several NRs play crucial roles in the regulation of hepatic lipid metabolism.^[^ ^12^ ^]^ For example, liver peroxisome proliferator activated receptor *α* (PPAR*α*) is essential for the utilization of FFAs and protective against NAFLD by induction of genes involved in peroxisomal and mitochondrial *β*‐oxidation.^[^ ^13^ ^]^ Liver X receptor (LXR) acts as a sterol sensor to enhance hepatic lipogenesis, due in part to its ability to promote the expression of SREBP‐1c and lipogenic genes.^[^ ^14^ ^]^ Conversely, farnesoid X receptor (FXR), a bile acid sensor, could inhibit lipogenesis through suppression of SREBP‐1c.^[^ ^14^ ^]^ Therefore, understanding the metabolic roles of NRs may shed more light on the development of novel therapies for treating NAFLD and related metabolic diseases.

Ob/ob (leptin deficient) and db/db (leptin receptor deficient) mice, and high‐fat‐diet (HFD) fed mice, are principal animal models to investigate the molecular mechanisms of NAFLD.^[^ ^2^ ^]^ However, these mice have many symptoms of metabolic diseases, such as obesity, polyphagia, and hyperglycemia, which may confound the study of regulatory events leading to liver steatosis. We and others previously demonstrated that the ubiquitous transcription factor Yin Yang 1 (YY1) is strongly associated with the development of NAFLD in animals and human subjects.^[^ ^15^, ^16^, ^17^, ^18^ ^]^ Forced overexpression of YY1 in the livers of lean mice functionally promotes hepatosteatosis without altering body weight and food intake, while knockdown of hepatic YY1 in db/db mice improves liver steatosis.^[^ ^15^ ^]^ Thus, we used these mice to explore differential expressed genes that contribute to NAFLD. As a result, Chicken ovalbumin upstream promoter transcription factor III (COUP‐TFIII) (also known as NR2F6, nuclear receptor subfamily 2, group F, member 6) was identified as a potential novel regulator of hepatic triglyceride homeostasis. The orphan nuclear receptors COUP‐TFI, COUP‐TFII, and COUP‐TFIII define a subfamily that participate in embryonic development, cell fate decisions, cell proliferation, and tumorigenesis.^[^ ^19^, ^20^ ^]^ It has been shown that COUP‐TFII is required for maintaining adipogenesis, lipid homeostasis, and energy expenditure.^[^ ^21^ ^]^ Nevertheless, the metabolic functions of other two COUP‐TFs remain uncovered. Our results showed that NR2F6 has a steatotic role in the development of obesity‐associated NAFLD and insulin resistance through direct upregulation of CD36 expression.

## 2 Results

### 2.1 Identification of NR2F6 as a Novel Target of Yin Yang 1

**Figure 1:**
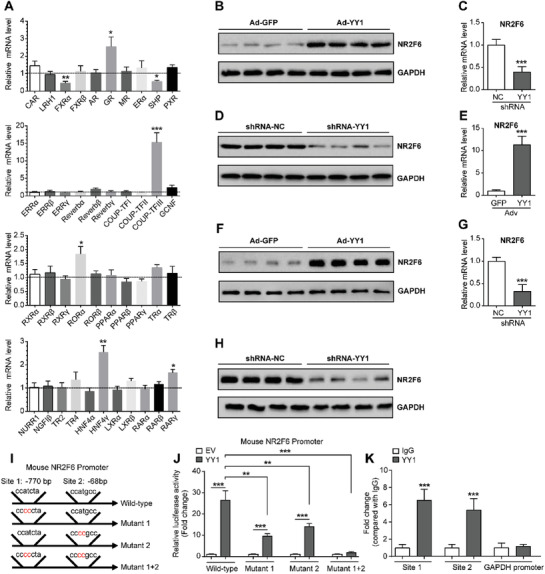
Identification of NR2F6 as a novel target of Yin Yang 1. A) Relative mRNA levels of 41 nuclear receptors in the livers of C57BL/6 mice. Mice were administrated with adenovirus containing GFP or Ying Yang 1 (YY1) through tail vein injection and sacrificed at day 10 post‐injection. Transcript levels were measured by quantitative real‐time PCR and normalized to Ad‐GFP (set as 1, dotted line). *n* = 5 in each group. B) Protein levels of NR2F6 in the livers of mice overexpressing GFP or YY1. C) Relative mRNA levels of NR2F6 in the livers of *db/db* mice. Mice were administrated with adenoviral YY1 shRNA or negative control (NC) through tail vein injection and sacrificed at day 12 post‐injection. *n* = 6 in each group. D) Protein levels of NR2F6 in the livers of *db/db* mice infected with adenoviral YY1 shRNA or negative control (NC). E) Relative mRNA levels of NR2F6 in mouse primary hepatocytes (MPHs) transfected with Ad‐GFP or Ad‐YY1 for 36 h. *n* = 4 in each group. F) Protein levels of NR2F6 in MPHs transfected with Ad‐GFP or Ad‐YY1 for 48 h. G) Relative mRNA levels of NR2F6 in MPHs transfected with adenoviral YY1 shRNA or negative control (NC) for 36 h. *n* = 4 in each group. H) Protein levels of NR2F6 in MPHs transfected with adenoviral YY1 shRNA or negative control (NC) for 48 h. I) The proximal promoter region of mouse NR2F6 gene contains two potential binding sites for YY1. J) Luciferase reporter assays. HepG2 cells were co‐transfected with YY1 expression plasmids and luciferase reporter plasmids containing wild‐type or mutant NR2F6 promoters. *n* = 5 in each group. K) Chromatin immunoprecipitation (ChIP) assays showing representative YY1 binding to the two sites of NR2F6 promoter region in MPHs. *n* = 5 in each group. Data are presented as mean ± SEM. 2‐tailed Student's *t*‐test (A, C, E, G, K). 1‐way ANOVA followed by the Student–Newman–Keuls test (J). :*p* < 0.05, ::*p* < 0.01, :::*p* < 0.001.

At the molecular level, our previous data found that YY1 can inhibit the expression of FXR in the hepatocytes.^[^ ^15^ ^]^ YY1 is known to have a fundamental role in initiating, activating, or repressing the transcription of many genes,^[^ ^22^ ^]^ including several nuclear receptors (NRs).^[^ ^15^, ^23^, ^24^ ^]^ We therefore examined whether other NRs could be regulated by YY1. Of the 41 NRs that are expressed in liver,^[^ ^25^ ^]^ a pronounced upregulation of COUP‐TFIII/NR2F6 was observed in C57BL/6 mice with YY1 overexpression by quantitative real‐time PCR (qRT‐PCR) (**Figure** **1**A), which was confirmed by western blots (Figure 1B). In contrast, knockdown of YY1 led to a reduced expression of NR2F6 in the livers of db/db mice (Figure 1C,D). The positive regulation of NR2F6 by YY1 was also observed in mouse primary hepatocytes (MPHs) and HepG2 cells (Figure 1E–H; Figure S1A–D, Supporting Information).

To elucidate the molecular basis for this regulation, the proximal promoter region (position −1000 bp from the transcription start site) of mouse NR2F6 gene was scanned using an online transcription factor scanning system (PROMO, http://alggen.lsi.upc.edu/recerca/menu_recerca.html). In this range, two YY1 potential binding sites were identified at −770 and −68 bp (Figure 1I), which are similar to YY1 core motifs as reported before.^[^ ^26^, ^27^ ^]^ We then generated a luciferase reporter containing this region and the results showed that transient YY1 overexpression could substantially enhance the transcriptional activity of the intact wild‐type promoter (Figure 1J). However, mutation in either or both sites partially or largely blocked the role of YY1 (Figure 1J). To validate the luciferase reporter results in a physiological condition, we performed chromatin immunoprecipitation (ChIP) experiments on MPHs. This assay revealed the direct binding of YY1 to both sites at the NR2F6 promoter (Figure 1K). Taken together, our findings indicate that NR2F6 is novel transcriptional target of YY1 in the hepatocytes.

### 2.2 NR2F6 Promotes TG Accumulation in the Liver

**Figure 2:**
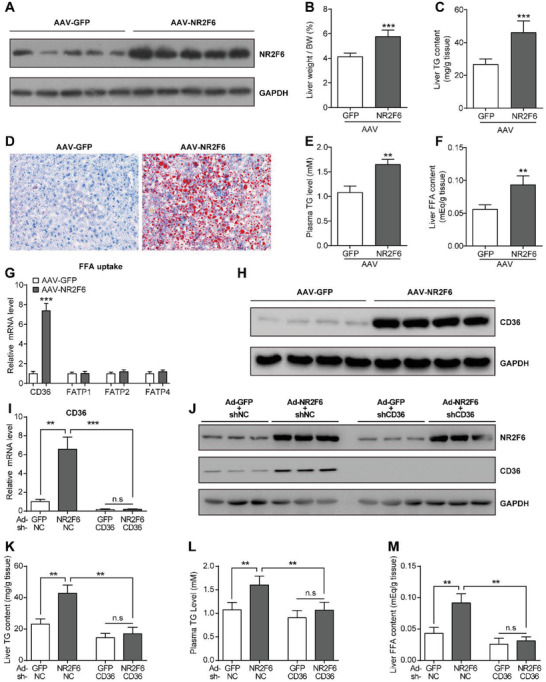
Overexpression of NR2F6 promotes liver steatosis in C57BL/6 mice. C57BL/6 mice were administrated with adeno‐associated virus (AAV9) containing GFP or NR2F6 through tail vein injection and sacrificed at day 30 post‐injection. *n* = 5 in each group. A) Protein levels of NR2F6 in the livers of mice. B) Liver weight, C) liver TG contents, D) representative Oil Red O staining of liver histology, E) plasma TG levels, and F) hepatic FFA concentrations in the mice. G) Relative mRNA levels of gene related to FFA uptake in the livers of mice. H) Protein expression of CD36 in the livers of mice. C57BL/6 mice were administrated with adenovirus virus (GFP or NR2F6) and shRNAs (CD36 or negative control) through tail vein injection and sacrificed at day 7 post‐injection. *n* = 5 in each group. I) Relative mRNA levels of CD36 in the liver. J) Protein levels of NR2F6 and CD36 in the liver. K) Liver TG contents, L) plasma TG levels, and M) hepatic FFA concentrations in the mice. Data are presented as mean ± SEM. 2‐tailed Student's *t*‐test (B, C, E–G). 1‐way ANOVA followed by the Student–Newman–Keuls test (I, K–M). :*p* < 0.05, ::*p* < 0.01, :::*p* < 0.001.

We next examined whether NR2F6 has metabolic effects in the livers. Thus, adeno‐associated virus (AAV9) containing an NR2F6 coding region (AAV‐NR2F6) or negative control (AAV‐green fluorescent protein, AAV‐GFP) was administrated into C57BL/6 mice via tail‐vein injection. The AAV9 used in this experiment employed a liver‐specific thyroxine‐binding globulin (TBG) promoter. This treatment led to an overexpression of NR2F6 in the livers (**Figure** **2**A), but not in other tissues examined (data not shown). Although body weight and food intake were not altered (Figure S2A,B, Supporting Information), liver weights and TG contents were significantly increased in mice with NR2F6 overexpression (Figure 2B,C), compared to control mice. The accumulation of hepatic lipids was also revealed by Oil Red O staining of liver sections (Figure 2D). Besides, plasma TG levels were increased in Ad‐NR2F6‐injected mice (Figure 2E), while plasma cholesterol levels remained unchanged (Figure S2C, Supporting Information). Therefore, NR2F6 overexpression in healthy mice predominantly induces hepatic TG accumulation.

Liver TG contents are determined by the balance of de novo lipogenesis, fatty acid uptake, fatty acid *β*‐oxidation, and very‐low‐density lipoprotein (VLDL) secretion.^[^ ^7^ ^]^ Our results showed that circulating levels of *β*‐hydroxybutyrate and the rate of hepatic VLDL secretion were not altered by NR2F6 overexpression (Figure S2D,E, Supporting Information). In contrast, hepatic FFA concentrations were significantly increased in mice overexpressing NR2F6 (Figure 2F).

To elucidate the mechanisms underlying the effects of NR2F6 on hepatic TG metabolism, RNA‐sequencing analysis was performed using livers of mice expressing AAV‐NR2F6 and AAV‐GFP, using *p* < 0.05 and fold change > 1.5 as the thresholds (Figure S3A, Supporting Information). We were particularly interested in the observed enrichment of the fatty acid transport pathway given the multitude of research indicating that it plays a crucial role in the hepatic TG metabolism (Figure S3B,C, Supporting Information). Subsequent qRT‐PCR confirmed that mRNA expression of CD36 was significantly induced in the livers of AAV‐NR2F6‐injected mice (Figure 2G). The upregulation of CD36 at the protein level was confirmed by western blots (Figure 2H). However, expression of other transporters, such as FATP1, FATP2, and FATP4, were unchanged (Figure 2G). Besides, expression levels of genes involved in de novo lipogenesis, fatty acid *β*‐oxidation, and lipid secretion remained unaltered or minorly changed (Figure S3D–F, Supporting Information). Recent studies showed that lipid droplet‐associated proteins, such as cell death‐inducing DNA fragmentation factor, alpha subunit‐like effector A (Cidea) and Cidec, play a critical role in hepatosteatosis.^[^ ^28^, ^29^ ^]^ However, their expression was not induced by YY1 or NR2F6 overexpression (Figure S3G,H, Supporting Information). Additionally, we introduced NR2F6 in the liver while simultaneously depleting CD36 with an adenovirus that expresses CD36‐targeting shRNA in C57BL/6 mice (Figure 2I,J). As a result, liver TG contents, plasma TG, and hepatic FFA levels were reduced in CD36‐depleted mice in the presence of NR2F6 overexpression (Figure 2K–M), suggesting that NR2F6 cannot promote liver steatosis in the absence of CD36. Thus, we speculate that forced expression of NR2F6 could promote liver steatosis by specifically enhancing FFA uptake through CD36.

### 2.3 NR2F6 Promotes CD36 Expression through Directly Binding to the CD36 Promoter

**Figure 3:**
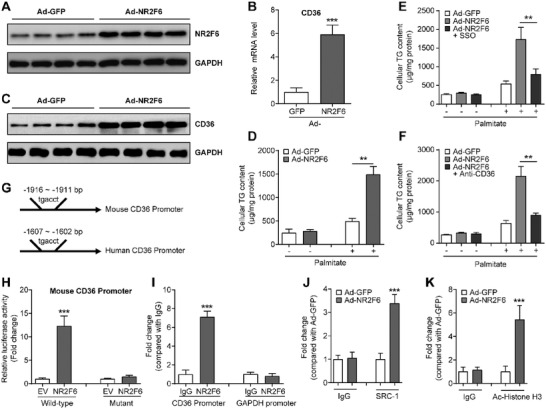
NR2F6 regulates CD36 expression. A) Protein levels of NR2F6 in MPHs transfected with Ad‐GFP or Ad‐NR2F6 for 48 h. B) Relative mRNA levels of CD36 in MPHs transfected with Ad‐GFP or Ad‐NR2F6 for 36 h. *n* = 4 in each group. C) Protein levels of CD36 in MPHs transfected with Ad‐GFP or Ad‐NR2F6 for 48 h. D) Cellular TG contents in MPHs. Cells were serum‐starved for 16 h and then treated with palmitate (0.2mm)  for another 24 h. *n* = 4 in each group. Cellular TG contents in MPHs. Cells were serum‐starved and pre‐incubated with E) SSO or F) anti‐CD36 antibody (2µg mL^−1^) for 4 h. Then, cells were treated with palmitate (0.2mm) for another 24 h. *n* = 4 in each group. G) Promoter region of CD36 contains a potential conserved binding site for NR2F6. H) Luciferase reporter assays. HepG2 cells were co‐transfected with NR2F6 expression plasmids and luciferase reporter plasmids containing wild‐type or mutant mouse CD36 promoters. *n* = 5 in each group. I) ChIP assays showing representative NR2F6 binding to the promoter region of mouse CD36 gene in MPHs. *n* = 5 in each group. ChIP assays showing J) the recruitment of SRC‐1 and K) status of acetylated histone H3 at the promoter region of mouse CD36 gene in MPHs. Cells were transfected with Ad‐GFP or Ad‐NR2F6 for 24 h and then subjected to ChIP assays. *n* = 4 in each group. Data are presented as mean ± SEM. 2‐tailed Student's *t*‐test (B, D, H–K). 1‐way ANOVA followed by the Student–Newman–Keuls test (E, F). ::*p* < 0.01, :::*p* < 0.001.

To confirm the in vivo findings, MPHs and HepG2 cells were transfected with adenovirus containing NR2F6 or GFP (**Figure** **3**A; Figure S3I, Supporting Information). As expected, elevated CD36 expression was observed in both cells transfected with Ad‐NR2F6 (Figure 3B,C; Figure S3I,J, Supporting Information). Although cellular TG contents were only slightly increased in cells overexpressing NR2F6 under serum starvation conditions, forced NR2F6 expression led to a substantial TG retention in the presence of palmitate (Figure 3D; Figure S3K, Supporting Information). However, pre‐incubation of a CD36 inhibitor (Sulfo‐*N*‐Succinimidyl Oleate, SSO),^[^ ^30^ ^]^ or a CD36 blocking antibody,^[^ ^31^ ^]^ largely attenuated Ad‐NR2F6‐induced cellular TG accumulation, further confirming the importance of CD36 in the steatotic role of NR2F6 (Figure 3E,F; Figure S3L,M, Supporting Information).

We next sought to determine whether CD36 could be a direct transcriptional target gene of NR2F6. Previous studies have demonstrated the NR2F‐specific binding oligonucleotide sequence contains a TGACCT motif.^[^ ^32^, ^33^ ^]^ We analyzed the promoter region of mouse CD36 gene (−2000 bp from the transcriptional start site) and found a potential NR2F6 binding site at −1916 bp (Figure 3G). It is of interest that this binding site also exists in the promoter region of human CD36 gene (Figure 3G). To test whether this site is functional and responsible for the regulatory role of NR2F6 on CD36 expression, luciferase reporter assays were performed. As a result, transient NR2F6 overexpression upregulated the promoter activity of mouse CD36 gene, whereas mutation of the binding site abolished the effects of NR2F6 (Figure 3H). Similar results were obtained using human CD36 gene promoter (Figure S3N, Supporting Information). Moreover, the binding affinity of NR2F6 to the CD36 promoter was also confirmed by ChIP experiments in MPHs (Figure 3I).

It has been well‐established that NRs usually promote gene transcription by recruiting several cofactors, such as SRC‐1, thereby facilitating histone modification on the promoter region of target genes.^[^ ^34^ ^]^ Our ChIP assays showed that NR2F6 overexpression could enhance the recruitment of SRC‐1 and appearance of acetylated Histone H3 at the NR2F6 binding region of CD36 promoter (Figure 3J,K), thereby facilitating its transcription. Together, these findings support a role for a direct regulatory function of NR2F6 in induction of CD36 transcriptional activation.

### 2.4 Hepatic NR2F6 Expression Is Increased in Mouse and Human Obesity

**Figure 4:**
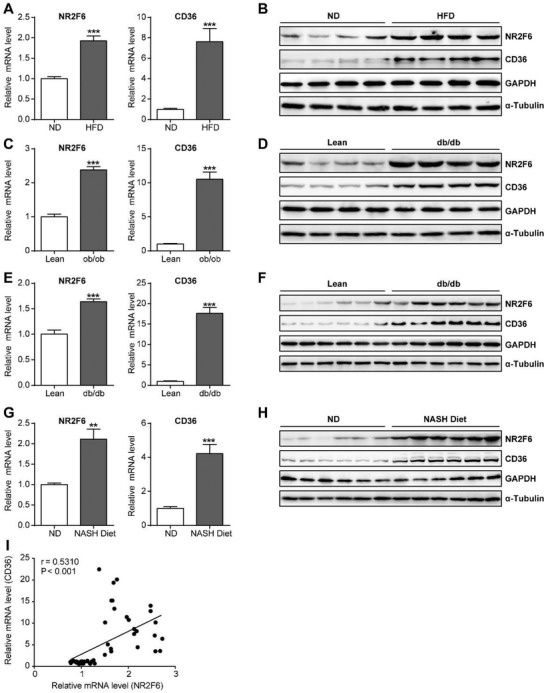
Upregulation of NR2F6 in the livers of mouse models of NAFLD. A) Relative mRNA levels and B) protein expression of NR2F6 and CD36 in the livers of C57BL/6 mice fed a normal diet or high fat diet for 12 weeks. *n* = 6 in each group. C) Relative mRNA levels and D) protein expression of NR2F6 and CD36 in the livers of lean and *ob/ob* mice. *n* = 5 in each group. E) Relative mRNA levels and F) protein expression of NR2F6 and CD36 in the livers of lean and *db/db* mice. *n* = 6 in each group. G) Relative mRNA levels and H) protein expression of NR2F6 and CD36 in the livers of C57BL/6 mice fed a normal diet or NASH diet for 12 weeks. *n* = 6 in each group. I) Pearson *r* and *p* values for normalized NR2F6 mRNA levels versus normalized CD36 mRNA levels in the mouse livers as indicated in (A–H) (*n* = 34 in total). Data are presented as mean ± SEM. 2‐tailed Student's *t*‐test (A, C, E, G). Pearson correlation analysis (I). ::*p* < 0.01, :::*p* < 0.001.

The steatotic role of NR2F6 promoted us to ask whether NR2F6 expression is altered in the livers of obese mice and human subjects. As a result, hepatic mRNA as well as protein levels of NR2F6 were significantly higher in mice fed a high‐fat‐diet (HFD) for 12 weeks, compared to that in C57BL/6 mice fed a normal chow diet (ND) (**Figure** **4**A,B). In agreement, the expression levels of NR2F6 were also found to be increased in livers of both ob/ob and db/db mice, compared to that in the corresponding lean mice (Figure 4C–F). However, expression levels of NR2F6 in while adipose tissue (WAT), brown adipose tissue (BAT), and skeletal muscle (SKM) were unaltered (Figure S4A–C, Supporting Information), suggesting that obesity‐associated upregulation of NR2F6 is liver‐specific. Consistently, mRNA and protein levels of CD36 were significantly increased in the livers of obese mice, compared to those in the corresponding control mice (Figure 4A–F). We also fed C57BL/6 mice with a recently described diet containing high‐fat and high‐fructose to induce NASH,^[^ ^35^, ^36^ ^]^ and found that NR2F6 and CD36 were also upregulated in the livers of these mice (Figure 4G,H). Pearson correlation analysis showed that mRNA levels of NR2F6 and CD36 are positively and significantly correlated in the mouse livers (Figure 4I).

**Figure 5:**
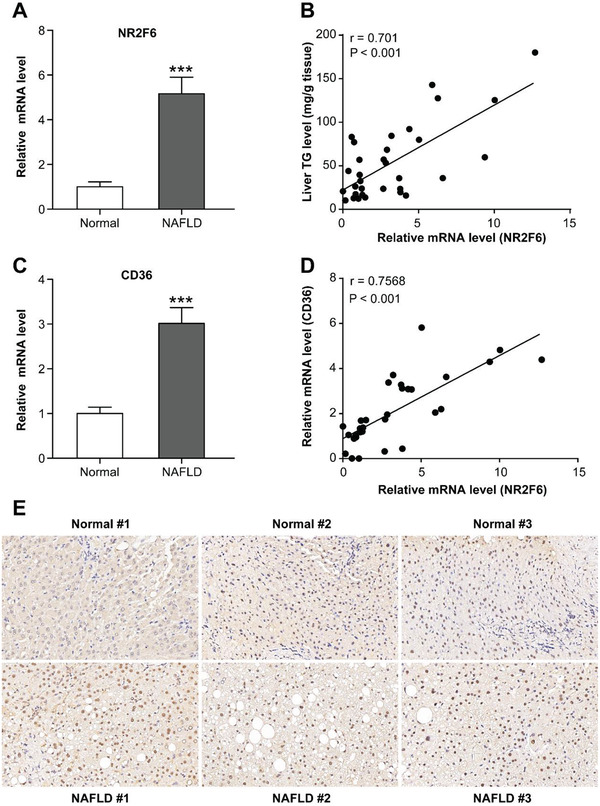
NR2F6 expression is increased in the livers of NAFLD patients. A) Relative mRNA levels of NR2F6 in livers from normal subjects (*n* = 15) and NAFLD patients (*n* = 17). B) Pearson *r* and *p* values for normalized NR2F6 mRNA levels versus TG contents in human livers (*n* = 32). C) Relative mRNA levels of CD36 in livers from normal subjects (*n* = 15) and non‐alcoholic fatty liver disease (NAFLD) patients (*n* = 17). D) Pearson *r* and *p* values for normalized NR2F6 mRNA levels versus normalized CD36 mRNA levels in human livers (*n* = 32). E) Representative immunohistochemistry staining of NR2F6 in liver sections from normal subjects and NAFLD patients. Magnification: ×400. Data are presented as mean ± SEM. 2‐tailed Student's *t*‐test (A,C). Pearson correlation analysis (B,D). :*p* < 0.05, ::*p* < 0.01, :::*p* < 0.001.

Moreover, we analyzed their mRNA expression in the livers of human patient cohort. Our results found that NR2F6 mRNA levels were increased in the patients with NAFLD than in the normal subjects (**Figure** **5**A). Moreover, the mRNA levels of NR2F6 were significantly and positively correlated with hepatic TG contents (Figure 5B). In agreement, CD36 mRNA expression was also increased in NAFLD patients and correlated with mRNA levels of NR2F6 (Figure 5C,D). The increased expression of NR2F6 was confirmed by immunohistochemistry (Figure 5E). Cellular experiments also revealed that NR2F6 expression could be upregulated by palmitate but not high glucose (Figure S4D–G, Supporting Information). Together with the observations that Ad‐NR2F6‐injected mice exhibited more hepatic TG contents, these findings suggest that upregulation of NR2F6 represents a common feature of obesity‐related dyslipidemia and might be causally linked to the pathogenesis of NAFLD.

### 2.5 Knockdown of NR2F6 Improves Hepatosteatosis and Insulin Resistance

**Figure 6:**
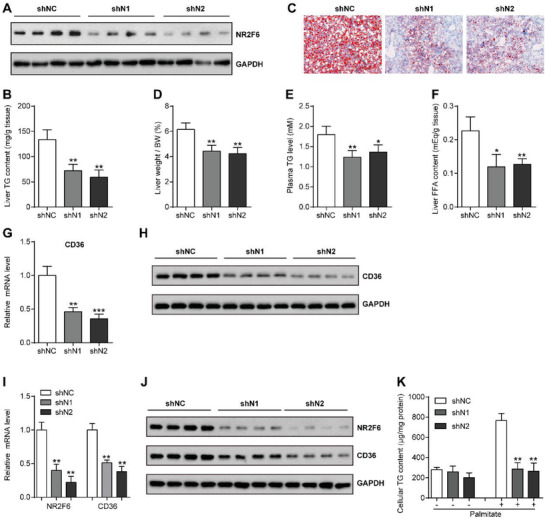
Liver‐specific suppression of NR2F6 improves obesity‐associated hepatosteatosis. *Db/db* mice were administrated with two adenoviral NR2F6 shRNAs (shN1, shN2) or negative control (shNC) through tail vein injection and sacrificed at day 14 post‐injection. *n* = 6 in each group. A) Representative protein levels of NR2F6 in the livers of *db/db* mice. B) Liver TG contents, C) representative Oil Red O staining of liver sections, D) liver weight, E) plasma TG levels, and F) hepatic FFA concentrations in *db/db* mice. G) Relative mRNA levels and H) protein expression of CD36 in the livers of mice. I) Relative mRNA levels and J) protein expression of CD36 in the MPHs transfected with shN1, shN2, or shNC. *n* = 6 in each group. K) Cellular TG contents in the MPHs. *n* = 4 in each group. Data are presented as mean ± SEM. 1‐way ANOVA followed by the Student–Newman–Keuls test (B,D–G,I,K). :*p* < 0.05, ::*p* < 0.01, :::*p* < 0.001.

Having established the efficiency of NR2F6 overexpression to induce hepatosteatosis, we next investigated whether repression of NR2F6 will reduce TG accumulation in the livers of obese mice. To test this, db/db mice were treated with two adenoviruses carrying small hairpin RNAs (shRNAs) targeting NR2F6 (Ad‐shN1, Ad‐shN2) or negative control (Ad‐shNC). Reduced protein contents of NR2F6 were detected in the livers of Ad‐shN1 and Ad‐shN2‐injected db/db mice, compared to that in the livers of Ad‐shNC‐injected mice (**Figure** **6**A). Food intake and body weight were not altered among three groups (Figure S5A,B, Supporting Information). However, measurement of the consequences on liver TG metabolism demonstrated that Ad‐shN1 and Ad‐shN2‐injected db/db mice had reduced hepatic TG contents (Figure 6B). Knockdown of NR2F6 considerably decreased the number and size of large lipid droplets in mouse livers as shown by Oil Red O staining (Figure 6C). In agreement, liver weight, plasma TG concentrations, and hepatic fatty acid levels were also lower in db/db mice depleted of NR2F6 (Figure 6D–F). The beneficial effects of NR2F6 deficiency was associated with a decrease in hepatic CD36 expression (Figure 6G–H), while mRNA levels of genes related to lipogenesis, *β*‐oxidation, and other fatty acid transporters remained unaltered (Figure S5C–E, Supporting Information). We also examined the effects of hepatic knockdown of NR2F6 in high‐fat‐diet‐induced obese mice. As with results observed in the db/db mice, HFD mice administrated with Ad‐shN1 and Ad‐shN2 displayed significantly reduced CD36 expression, liver and plasma TG contents, and hepatic FFA concentrations (Figure S6A–F, Supporting Information). Moreover, consistent with the in vivo findings, knockdown of NR2F6 also suppressed CD36 expression and improved palmitate‐induced TG accumulation in MPHs (Figure 6I–K). Together, all of these results demonstrate that hepatocyte‐specific inhibition of NR2F6 can improve obesity‐associated liver steatosis.

The development of liver steatosis and insulin resistance are usually strongly associated.^[^ ^1^, ^2^, ^3^ ^]^ Besides, increased CD36 expression in the livers has been associated with insulin resistance and hyperinsulinemia,^[^ ^37^ ^]^ while liver‐specific ablation of CD36 improves insulin sensitivity in HFD‐fed mice.^[^ ^38^ ^]^ Therefore, we speculate that overexpression of NR2F6 might disrupt glucose homeostasis in lean mice, while knockdown of NR2F6 could improve whole‐body glucose homeostasis in obese mice. As expected, the NR2F6‐expressing C57BL/6 mice had significantly increased blood glucose levels and plasma insulin levels compared to control mice (Figure S7A,B, Supporting Information). Besides, glucose tolerance test (GTT) and insulin tolerance test (ITT) showed a worsen glucose tolerance and reduced insulin sensitivity in the NR2F6‐expressing group (Figure S7C,D, Supporting Information). On the other hand, Ad‐shN1 and Ad‐shN2‐injected db/db and HFD mice had lower blood glucose and plasma insulin concentrations, compared with Ad‐shNC‐injected control mice (Figure S8A–D, Supporting Information). In agreement, insulin tolerance tests (ITTs) revealed a significant enhanced insulin sensitivity in the Ad‐shN1‐ and Ad‐shN2‐treated mice (Figure S8E,F, Supporting Information).

### 2.6 Knockdown of NR2F6 Attenuates MCD‐Diet‐Induced Steatohepatitis

**Figure 7:**
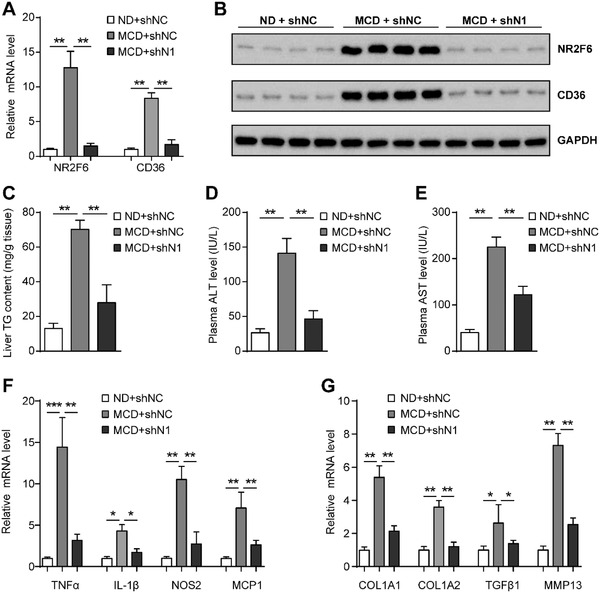
NR2F6 deficiency ameliorated MCD‐induced liver injury. C57BL/6 mice were fed with a methionine and choline‐deficient (MCD) diet for 6 weeks and then administrated with Ad‐shN1 or Ad‐shNC through tail vein injection. Mice were sacrificed at day 14 post‐injection. *n* = 5 in each group. A) Relative mRNA levels and B) protein expression of CD36 and NR2F6 in the livers of mice. C) Liver TG contents, D,E) plasma ALT and AST levels in mice. Relative mRNA levels of genes involved in F) hepatic inflammation and G) fibrosis. Data are presented as mean ± SEM. 1‐way ANOVA followed by the Student–Newman–Keuls test (A,C–G). :*p* < 0.05, ::*p* < 0.01, :::*p* < 0.001.

CD36 has been shown to participate in the pathogenesis of NASH,^[^ ^39^, ^40^ ^]^ and we also found that its expression is regulated in the livers of mice fed a NASH diet (Figure 4C). We therefore test the role of NR2F6 in NASH. C57BL/6 mice were fed with a methionine and choline‐deficient (MCD) diet for 6 weeks and then administrated with Ad‐shN1 or Ad‐shNC. Both NR2F6 and CD36 were strongly induced in mice fed with an MCD diet (**Figure** **7**A,B). However, MCD‐induced expression of NR2F6 and CD36 was successfully blocked by Ad‐shN1 (Figure 7A,B). As a result, hepatic TG contents were significantly increased in mice fed an MCD diet (Figure 7C). However, ablation of hepatic NR2F6 gene markedly attenuated MCD diet‐induced hepatic TG retention (Figure 7C). In agreement, plasma levels of ALT and AST were reduced in mice expressing Ad‐shN1 (Figure 7D,E). Moreover, knockdown of NR2F6 reduced MCD diet‐associated expression of pro‐inflammatory and fibrosis‐related genes (Figure 7F,G). Thus, our findings suggest that suppression of NR2F6 could alleviate symptoms of MCD diet‐induced NASH, including liver steatosis, inflammation, and injury.

### 2.7 Effects of NR2F6 Expression in Mice on Metformin Action

**Figure 8:**
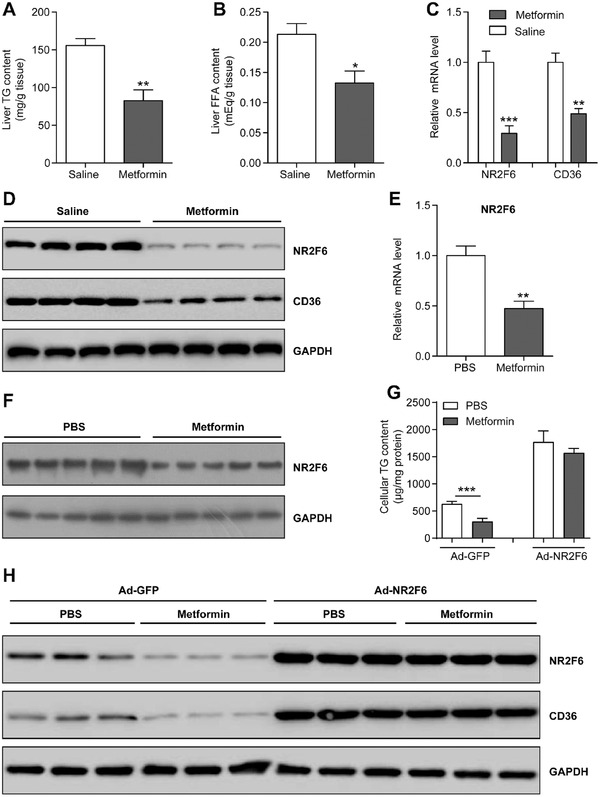
Metformin inhibits NR2F6 expression to attenuate hepatic steatosis. *Db/db* mice were daily treated with metformin (50 mg kg^−1^ per day) or vehicle control (saline) via intraperitoneal injection for 6 weeks. *n* = 5 in each group. A) Liver TG contents and B) FFA concentrations in two groups of *db/db* mice. C) Relative mRNA levels and D) protein expression of CD36 and NR2F6 in the livers of mice. *n* = 5 in each group. E) Relative mRNA levels and F) protein expression of NR2F6 in MPHs treated with Metformin (0.5mm) or vehicle control (PBS). *n* = 5 in each group. G) Cellular TG contents in MPHs. Cells were transfected with Ad‐GFP or Ad‐NR2F6 and incubated with palmitate (0.2 mm). *n* = 4 in each group. H) Protein expression levels of NR2F6 and CD36 in MPHs. Data are presented as mean ± SEM. 2‐tailed Student's *t*‐test (A–C,E,G). :*p* < 0.05, ::*p* < 0.01, :::*p* < 0.001.

Lots of animal experiments have demonstrated that the anti‐diabetic drug metformin can be used for the treatment of NAFLD,^[^ ^41^, ^42^ ^]^ which is also recapitulated in several clinical studies.^[^ ^43^, ^44^ ^]^ However, the mechanism of its anti‐steatotic action is still not well understood. Previous studies have shown that metformin treatment could downregulate CD36 expression in pancreatic *β* cells and skeletal muscles.^[^ ^45^, ^46^ ^]^ We therefore examine whether NR2F6/CD36 axis is the molecular target for the lipid‐lowering effects of metformin. To test it, db/db mice were administrated with metformin at a dose of 50 mg kg^−1^ for 4 weeks. This dose has been shown to improve liver steatosis in obese mice before.^[^ ^41^, ^42^ ^]^ As expected, treatment of metformin led to a reduction of hepatic TG and FFA contents (**Figure** **8**A,B). Interestingly, expression levels of NR2F6 and CD36 in the livers were significantly decreased by metformin (Figure 8C,D). Suppression of NR2F6 was confirmed in MPHs treated with metformin (Figure 8E,F).

It has been demonstrated that nuclear factor kappa B (NF‐*κ*B)/P65 is a critical regulator of YY1 transcription in multiple cell types.^[^ ^47^ ^]^ Our previous data also revealed that activation of NF‐*κ*B signaling pathway contributes to YY1 upregulation in obese livers.^[^ ^15^ ^]^ Considering that NR2F6 is a transcriptional target of YY1 (Figure 1), we examined whether metformin alters NR2F6 expression through NF‐*κ*B/YY1. Consistent with previous reports that the inhibition of NF‐*κ*B is one of the key mediators of metformin's effect,^[^ ^48^ ^]^ we found that metformin treatment reduced the phosphorylated P65 levels in the livers of db/db mice (Figure S9A, Supporting Information), subsequently leading to a decreased expression of YY1 (Figure S9A,B, Supporting Information). The reduction of phosphorylated P65 and YY1 by metformin was confirmed in MPHs (Figure S9C,D, Supporting Information). Consistently, ChIP assays revealed that metformin treatment decreased the binding and recruitment of YY1 onto the two sites of NR2F6 promoter region (Figure S9E, Supporting Information). Therefore, we speculate that metformin inhibits NR2F6 expression, at least in part, through suppressing NF‐*κ*B/ YY1 signaling pathway.

To explore further whether suppression of NR2F6 is responsible for the anti‐steatotic role of metformin, MPHs were transfected with Ad‐NR2F6 or Ad‐GFP, and then incubated with palmitate or palmitate plus metformin. As a result, NR2F6 overexpression largely abrogated the ability of metformin to decrease cellular TG contents and inhibit CD36 expression (Figure 8G,H). These data suggest that suppression of NR2F6 and CD36, may play a role in the metformin‐mediated improvement of hepatosteatosis.

## 3 Discussion

A hallmark of NAFLD is the presence of excessive TG in the hepatocytes due to imbalance of fatty acid uptake, synthesis, oxidation, and export.^[^ ^7^ ^]^ Interestingly, these processes in the liver are tightly controlled by several NRs and the abnormal dysfunction of NRs has been associated with hepatic steatosis.^[^ ^12^ ^]^ More importantly, NRs have become potential therapeutic targets of NAFLD and related metabolic diseases. For instance, several clinical studies have shown the efficacy of obeticholic acid (OCA), an FXR agonist, in the improvement of insulin sensitivity, liver histology, hepatic inflammation, and fibrosis.^[^ ^49^, ^50^, ^51^ ^]^ In this study, we for the first time, to our knowledge, uncovered an NR2F6/CD36 axis that plays a role for obesity‐associated NAFLD. NR2F6 overexpression promotes hepatic TG accumulation in healthy mice, while knockdown of NR2F6 improved hepatosteatosis in obese mice and MCD‐diet induced NASH mice. Notably, our findings showed that NR2F6 is aberrantly upregulated in the livers of obese mice, which is recapitulated in NAFLD patients. As such, identification of NR2F6 antagonists may provide a new therapeutic strategy for reversing or treating NAFLD/NASH pathogenesis.

At the mechanistic level, NR2F6 transcriptionally activate CD36 gene expression to promote fatty acid uptake and induce TG retention. CD36 is a transmembrane glycoprotein expressed in many metabolic organs, including the liver. The crucial role of this receptor in facilitating the uptake of fatty acids has been extensively investigated. In consistency, CD36 expression is markedly elevated in the livers of obese mice and NAFLD patients.^[^ ^10^, ^39^, ^40^, ^52^, ^53^ ^]^ Moreover, hepatic CD36 upregulation is significantly correlated with liver fat content and insulin resistance in patients with NASH.^[^ ^54^ ^]^ The significance of CD36 in steatosis support the notion that hepatic FFAs uptake is an important pathogenic factor contributing to NAFLD and NASH. However, the physiological and pathophysiological regulators of CD36 expression remain largely unknown. It has been shown that CD36 is a common target of LXR, pregnane X receptor (PXR), and PPAR*γ* at the transcriptional level.^[^ ^55^ ^]^ However, expression of PXR, LXR*α*, LXR*β*, and PPAR*γ* were not altered in the livers overexpressing YY1 (Figure 1A). Besides, expression levels of these nuclear receptors were not affected by NR2F6 overexpression or knockdown (Figure S10A,B, Supporting Information). Therefore, we speculate that upregulation of CD36 by NR2F6 may be independent of other nuclear receptors. In addition, recent studies suggest that inflammatory stress increases hepatic CD36 protein level via activation of the mTOR pathway to enhance its translational efficiency.^[^ ^56^ ^]^ Moreover, CD36 was highly palmitoylated in mice with NASH and palmitoylation was shown to help it translocate to the plasma membrane of hepatocytes, thereby facilitating fatty acid uptake.^[^ ^40^ ^]^ Therefore, together with these studies, our data suggest that the expression, translocation, and activity of CD36 are rather complicated in the progression of chronic liver disease. Further investigation is still needed to determine the identity of unknown factors that regulate CD36 expression and activity.

Several limitations in our study should be pointed out. First, although AAV or adenovirus‐mediated overexpression or knockdown has been widely used in the field of liver research, future work using liver‐specific knockout mice will further validate the role of long‐term NR2F6 depletion in the development of NAFLD and NASH, especially under the conditions of different metabolic status. Second, the role of NRs in the regulation of glucose and lipid metabolism is diverse, due to plenty of direct and indirect downstream target genes. Although we did not find any changes in the mRNA levels of genes related to TG synthesis and oxidation in the livers with NR2F6 overexpression or knockdown, whether NR2F6 can regulate these processes remains to be determined. However, we could not exclude the possibility that NR2F6 may regulate hepatic lipid metabolism by other mechanisms, in addition to increasing CD36 expression. Chromatin immunoprecipitation sequencing (ChIP‐seq) might help to explore more downstream target genes for NR2F6 in the liver. Additionally, NRs have been shown to regulate the inflammatory process, which is an important component in the development of NAFLD/NASH and insulin resistance. Given that NR2F6 is a critical regulator of immune response and cytokine production,^[^ ^32^, ^33^ ^]^ we speculate that the role of NR2F6 in the hepatic inflammation and lipid metabolism might be partially interdependent.

## 4 Conclusion

The present study demonstrated that NR2F6 is an important regulator of hepatic triglyceride homeostasis. Obesity‐induced upregulation of NR2F6 promotes the expression of CD36 in the liver, thereby facilitating fatty acid uptake and triglyceride retention, contributing to NAFLD and insulin resistance. Our findings might provide a new molecular basis for liver steatosis and identify NR2F6 as a therapeutic target for treatment of NAFLD/NASH and related liver diseases.

## 5 Experimental Section

##### Animal Experiments

Male C57BL/6J mice aged 8 weeks were purchased from the Shanghai Laboratory Animal Company (SLAC, Shanghai, China). ob/ob and db/db mice were obtained from Nanjing Biomedical Research Institute of Nanjing University (NBRI, Nanjing, China). For high fat diet feeding, C57BL/6J mice were fed a diet containing 60% fat, 20% carbohydrate, and 20% protein (D12492, Research Diets Inc.) for 12 weeks. For NASH diet feeding, C57BL/6J mice were fed a diet containing 40% fat, 22% fructose, and 2% cholesterol (D09100310, Research Diets Inc.) for 12 weeks. For the metformin treatment, db/db mice were administrated with metformin (50 mg kg^−1^ per day, Sigma‐Aldrich) or vehicle control (saline) via intraperitoneal injection for 6 weeks. The animal protocol was reviewed and approved by the Animal Care Committees of Shanghai Jiao Tong University School of Medicine.

##### Human Samples

For analysis of hepatic gene expression and triglyceride content in humans, the liver tissues were collected in subjects who donated their partial livers for liver transplantation.^[^ ^57^, ^58^ ^]^ The clinical characteristics of human subjects have been described previously.^[^ ^58^ ^]^ The human study was approved by the Human Research Ethics Committee of Zhongshan Hospital, Fudan University (approval number: Y2017‐134). Written informed consent was obtained from all subjects and the experiments were conducted according to the principles outlined in the Declaration of Helsinki.

##### Adeno‐Associated Virus and Adenovirus

For generation of liver‐specific overexpression of NR2F6, the NR2F6 coding sequence was cloned into an AAV plasmid (TBG‐AAV2.1) to create TBG‐NR2F6 vector, in which TBG promoter controls hepatocyte‐specific expression of NR2F6. Adenovirus‐producing plasmids using CMV promoter were constructed using adenoviral expression kit from Life Technologies. The adenovirus shuttle plasmid and helper plasmid were transfected into 293A producer cells in 6‐well‐plates. The media was replaced with DMEM containing 10% FBS and 1% penicillin/streptomycin 6 h later. The culture media was replaced with fresh media every 2–3 days until cytopathic effect (CPE) was observed. The cells were collected when 80% CPE was observed and adenovirus was harvested by freezing at −80 °C and thawing at 37 °C and repeating it for four times. Then cell lysates were removed by centrifugation at 8000 g for 5 min at 4 °C and the supernatant containing adenovirus particles was stored at −80 °C. To silence endogenous NR2F6 expression, db/db and HFD mice were administrated with two short hairpin (sh) RNA adenoviruses that target NR2F6. The two shRNAs designed to knockdown NR2F6 had the following target sequences: shN1, 5′‐GGGACAAGTCCAGTGGAAAGCATTA‐3′, and shN2, 5′‐CATCCCTCATCTCCCAGCTCTTCTT‐3′. The CD36 shRNA had a sequence of 5′‐GCTATTGCGACATGATTAA‐3′. The negative control shRNA virus (shNC) had a core‐scrambled non‐targeted sequence of 5′‐TTCTCCGAACGTGTCACGT‐3′.

##### Glucose and Insulin Tolerance Tests

For glucose tolerance tests, mice were given intraperitoneal injection of D‐glucose (2.0 mg g^−1^, Sigma‐Aldrich) after a 16‐h fast. For insulin tolerance tests, mice were injected with regular human insulin (Eli Lilly, 0.75 units per kg) after a 6‐h fast. Blood glucose was determined using a portable blood glucose meter (LifeScan).

##### Triglyceride and Cholesterol Quantification

Liver tissues were harvested and homogenized in 5% NP‐40 solution and heated up to 100 °C and then cooled down to room temperature. The tissue homogenates were centrifuged, and the supernatants were processed for measuring triglyceride and cholesterol contents using commercial kits (BioVision, Milpitas, USA).

##### Quantitative Real‐Time PCR

Total RNA was isolated from cell lysates or mouse liver tissues using the standard TRIzol method according to the manufacturer's instructions (Invitrogen). First‐strand cDNA was synthesized from each RNA sample using the Reverse Transcription System (Promega). Oligo dT was used to prime cDNA synthesis. To analyze gene expression, quantitative real‐time PCR was performed using an SYBR Green Premix Ex Taq (Takara Biotechnology) on LightCycler 480 (Roche, Basel, Switzerland). Relative quantification analysis of gene expression data was calculated according to the 2^−ΔΔ^ *^Ct^* method. The results of relative expression were normalized to mRNA levels of housekeeping gene *Rplp0*. Sequences of primers are shown in Table S1, Supporting Information.

##### Western Blotting

Proteins from tissues or cells were harvested using radioimmunoprecipitation buffer containing Tris‐HCl (50 mmol L^−1^), NaCl (150 mmol L^−1^), MgCl_2_ (5 mmol L^−1^), EDTA (2 mmol L^−1^), NaF (1 mmol L^−1^), 1% NP40, and 0.1% SDS. The protein concentrations were quantified using commercial kits (Pierce). All protein samples were equally subjected to 10% SDS polyacrylamide gels, transferred to polyvinylidene difluoride membranes by electrophoresis, incubated with primary and secondary antibodies, and finally visualized by a chemiluminescence detection kit (Millipore). The following primary antibodies were purchased: NR2F6 (Abcam, Catalog number: ab137496; R&D Company, Catalog number: PP‐N2025‐00), CD36 (Abcam, Catalog number: ab133625), GAPDH (Abcam, Catalog number: ab181602), phosphorylated P65 (Beyotime, Catalog number: AF5881), total P65 (Beyotime, Catalog number: AF0246), a‐Tubulin (Sigma‐Aldrich, Catalog number: T6199).

##### Plasmids Construction and Luciferase Reporter Assays

Flag‐tagged full‐length mouse NR2F6 was obtained by PCR amplification of the cDNA from mouse liver and cloned into pDC316 with NheI/NotI restriction sites. The 5′ ends of the mouse NR2F6 and CD36 gene extending from position −1000 bp (relative to the transcription start site) to +40 and position −2000 bp to +50 were cloned into the pGL4.12 (Promega) luciferase reporter plasmid with the XhoI/HindIII restriction sites, respectively. The primers used for plasmids construction are shown in Table S2, Supporting Information. For the luciferase reporter assays, transfection experiments were performed on HepG2 cells at 70–80% confluence using Lipofectamine 3000 (Invitrogen). Cells were harvested 36 h after transfection, and luciferase activity was measured using the Dual Luciferase Reporter Assay System (Promega).

##### Chromatin Immunoprecipitation Assays

ChIP experiments in mouse primary hepatocytes were performed using commercial kits following the manufacturer's protocols (Millipore). In brief, cells were fixed with formaldehyde and DNA was sheared to fragments at 200–1000 bp using sonication. Chromatin was incubated and precipitated with antibodies against YY1 (Santa Cruz Company, Catalog number: sc‐7341), NR2F6 (R&D Company, Catalog number: PP‐N2025‐00), SRC1 (Abcam Company, Catalog number: ab2859), or IgG. The acetylation status of histone H3 was analyzed by Acetyl‐Histone H3 ChIP Kit (Epigentek Company, Catalog number: P‐2012).

##### Statistical Analysis

All values were shown as the mean ± standard error of the mean (SEM). Statistical analysis was performed using GraphPad prism 6 software (GraphPad Inc., San Diego, CA, USA). A 2‐tailed unpaired Student's *t*‐test was performed to compare between two groups. 1‐way ANOVA followed by the Student–Newman–Keuls test was used to compare more than two groups. Correlation analysis was performed using the Pearson correlation test. *p* values less than 0.05 were considered statistically significant. :, ::, and ::: represent *p* < 0.05, *p* < 0.01, and *p* < 0.001, respectively.

## Conflict of Interest

The authors declare no conflict of interest.

## Supporting information

Supporting Information
