# Supplementary material for: The Nuclear Orphan Receptor NR2F6 Promotes Hepatic Steatosis through Upregulation of Fatty Acid Transporter CD36
Source: Adv Sci (Weinh). 2020 Sep 21;7(21):2002273. doi: 10.1002/advs.202002273 (PMC7610302; doi:10.1002/advs.202002273)
Supplement: Supplementary file 1 — Supporting Information [file ADVS-7-2002273-s001.pdf]

© 2020 Wiley-VCH GmbH

ADVANCED SCIENCE
Open Access

Supporting Information

for *Adv. Sci.,* DOI: 10.1002/advs.202002273

The Nuclear Orphan Receptor NR2F6 Promotes Hepatic Steatosis through Upregulation of Fatty Acid Transporter CD36

Bing Zhou, Lijing Jia, Zhijian Zhang, Liping Xiang, Youwen Yuan, Peilin Zheng, Bin Liu, Xingxing Ren, Hua Bian, Liwei Xie, Yao Li, Jieli Lu, Huijie Zhang, and Yan Lu**

Supporting Information

The Nuclear Orphan Receptor NR2F6 Promotes Hepatic Steatosis through Upregulation of Fatty Acid Transporter CD36

*Bing Zhou, Lijing Jia, Zhijian Zhang, Liping Xiang, Youwen Yuan, Peilin Zheng, Bin Liu, Xingxing Ren, Hua Bian, Liwei Xie, Yao Li, Jieli Lu, Huijie Zhang**, and *Yan Lu**

Supplementary Figures

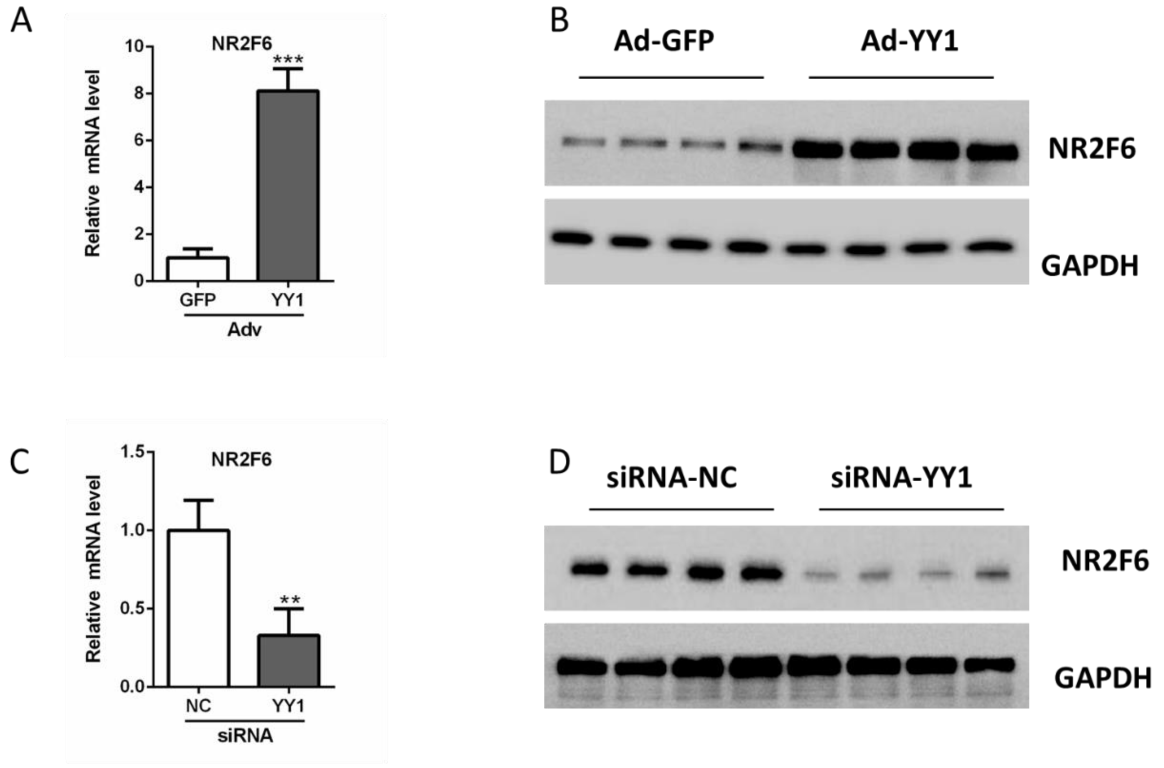

Figure S1

Figure S1. YY1 regulates NR2F6 expression in hepatocytes. A) Relative mRNA levels of NR2F6 in HepG2 cells transfected with Ad-GFP or Ad-YY1 for 36 hr. n=4 in each group. B) Protein levels of NR2F6 in HepG2 cells transfected with Ad-GFP or Ad-YY1 for 48 hr. C) Relative mRNA levels of NR2F6 in HepG2 cells transfected with siRNA oligos targeting NR2F6 or negative control (NC) for 36 hr. n=4 in each group. D) Protein levels of NR2F6 in HepG2 cells transfected with siRNA

oligos targeting NR2F6 or negative control (NC) for 48 hr. Data are presented as mean ± SEM. 2-tailed Student's t test (A, C). ** $P < 0.01$, *** $P < 0.001$.

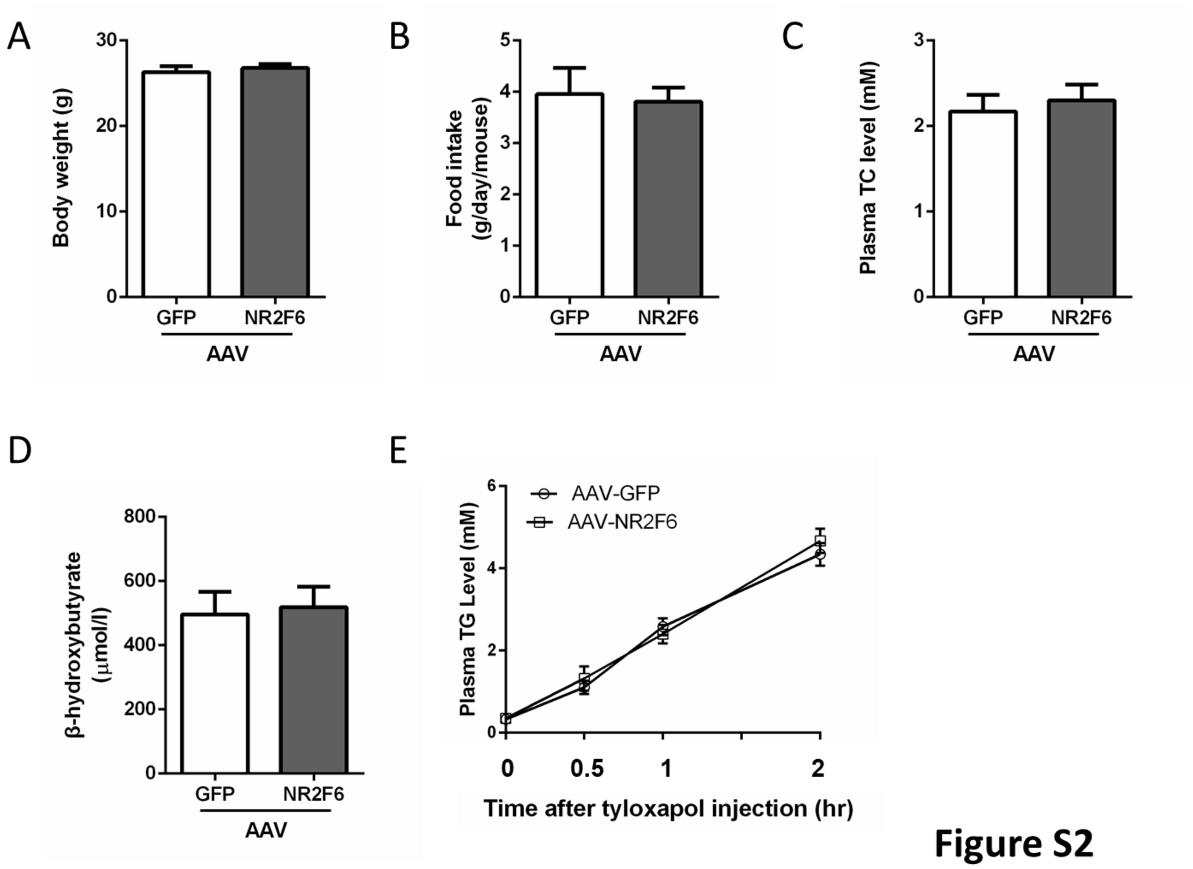

Figure S2

Figure S2. Metabolic phenotypes in C57BL/6 mice overexpressing NR2F6 or GFP. A-D) Body weight (A), food intake (B), plasma TC levels (C), plasma β-hydroxybutyrate levels (D) and in the mice overexpressing GFP or NR2F6. E) Increase in plasma TG following intravenous injection of tyloxapol (500 mg/kg) in two groups of mice. n=5 in each group. Data are presented as mean ± SEM. 2-tailed Student's t test (A-D). 1-way ANOVA followed by the Student-Newman-Keuls test (E).

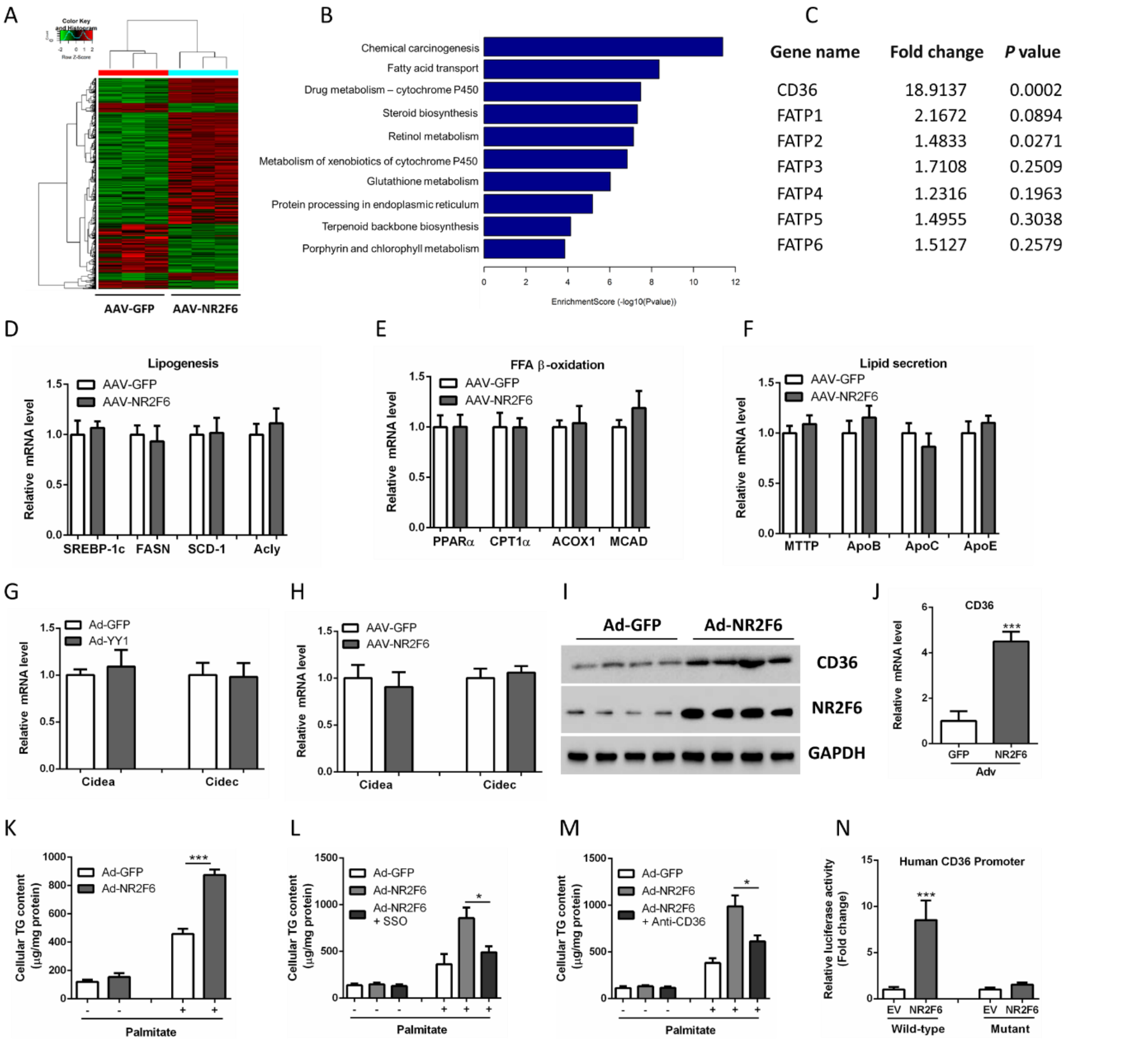

Figure S3

Figure S3. Upregulation of CD36 by NR2F6. A) Heatmap displaying the fold change in mRNA expression, as measured by RNA sequencing analysis of livers from mice infected with AAV-NR2F6 and AAV-GFP. B) Pathway analysis on the subset of genes upregulated >1.5 fold in the liver expressing AAV-NR2F6. C) RNA-Seq analysis showed the expression of fatty acid transporters. D-F) Relative mRNA levels of genes related to lipogenesis (D), FFA β-oxidation (E), and lipid secretion (F) in the livers of mice expressing AAV-NR2F6 or AAV-GFP. n=5 in each group. G) Relative mRNA levels of lipid droplet-associated genes in the livers of mice expressing Ad-YY1 or Ad-GFP. n=5 in each group. H) Relative mRNA levels of lipid droplet-associated genes in the

livers of mice expressing AAV-NR2F6 or AAV-GFP. n=5 in each group. I) Protein levels of NR2F6 and CD36 in HepG2 cells transfected with Ad-GFP or Ad-NR2F6 for 48 hr. J) Relative mRNA levels of CD36 in HepG2 cells transfected with Ad-GFP or Ad-NR2F6 for 36 hr. n=4 in each group. K) Cellular TG contents in HepG2 cells expressing Ad-NR2F6 or Ad-GFP in the absence or presence of palmitate (0.2mM). n=4 in each group. L-M) Cellular TG contents in HepG2 cells. Cells were serum-starved and pre-incubated with SSO (L) or anti-CD36 antibody (M, 2μg/ml) for 4 hr. Then, cells were treated with palmitate (0.2mM) for another 24 hr. n=4 in each group. N) Luciferase reporter assays. HepG2 cells were co-transfected with NR2F6 expression plasmids and luciferase reporter plasmids containing wild-type or mutant human CD36 promoters. n=4 in each group. Data are presented as mean ± SEM. 2-tailed Student's t test (D-H, J, K, N). 1-way ANOVA followed by the Student-Newman-Keuls test (L, M). * $P < 0.05$, *** $P < 0.001$.

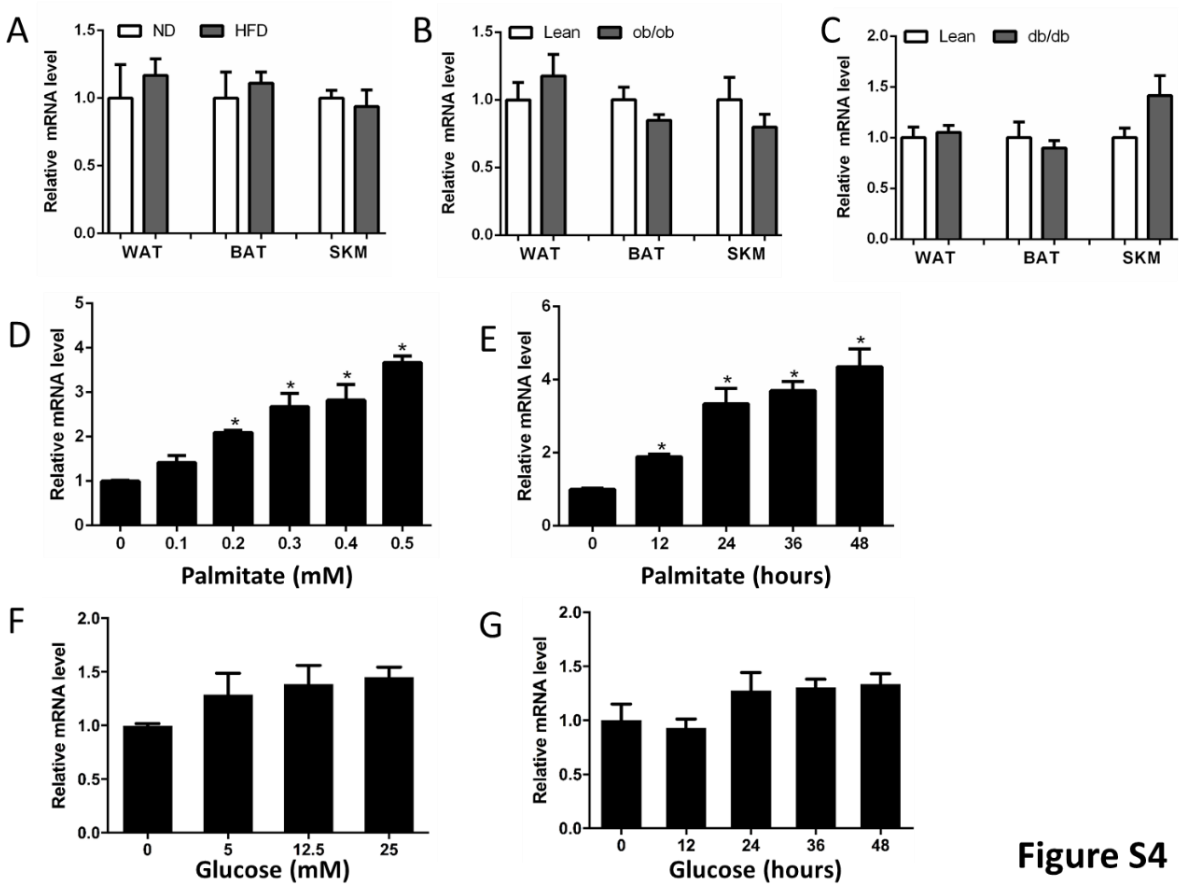

Figure S4. Expression of NR2F6 in obesity or by nutrients. A-C) Relative mRNA levels of NR2F6 in the white adipose tissue (WAT), brown adipose tissue (BAT) and skeletal muscles (SKM)

of HFD (A), *ob/ob* (B) and *db/db* (C) mice. n=5 in each group. D) Relative mRNA levels of NR2F6 in HepG2 cells treated with different dose of palmitate for 24 hr. n=3 in each group. E) Relative mRNA levels of NR2F6 in HepG2 cells treated with palmitate (0.3mM) for different time. n=3 in each group. F) Relative mRNA levels of NR2F6 in HepG2 cells treated with different dose of glucose for 24 hr. n=3 in each group. G) Relative mRNA levels of NR2F6 in HepG2 cells treated with high glucose (25mM) for different time. n=3 in each group. Data are presented as mean ± SEM. 2-tailed Student's t test (A-C). 1-way ANOVA followed by the Student-Newman-Keuls test (D-G). * $P < 0.05$.

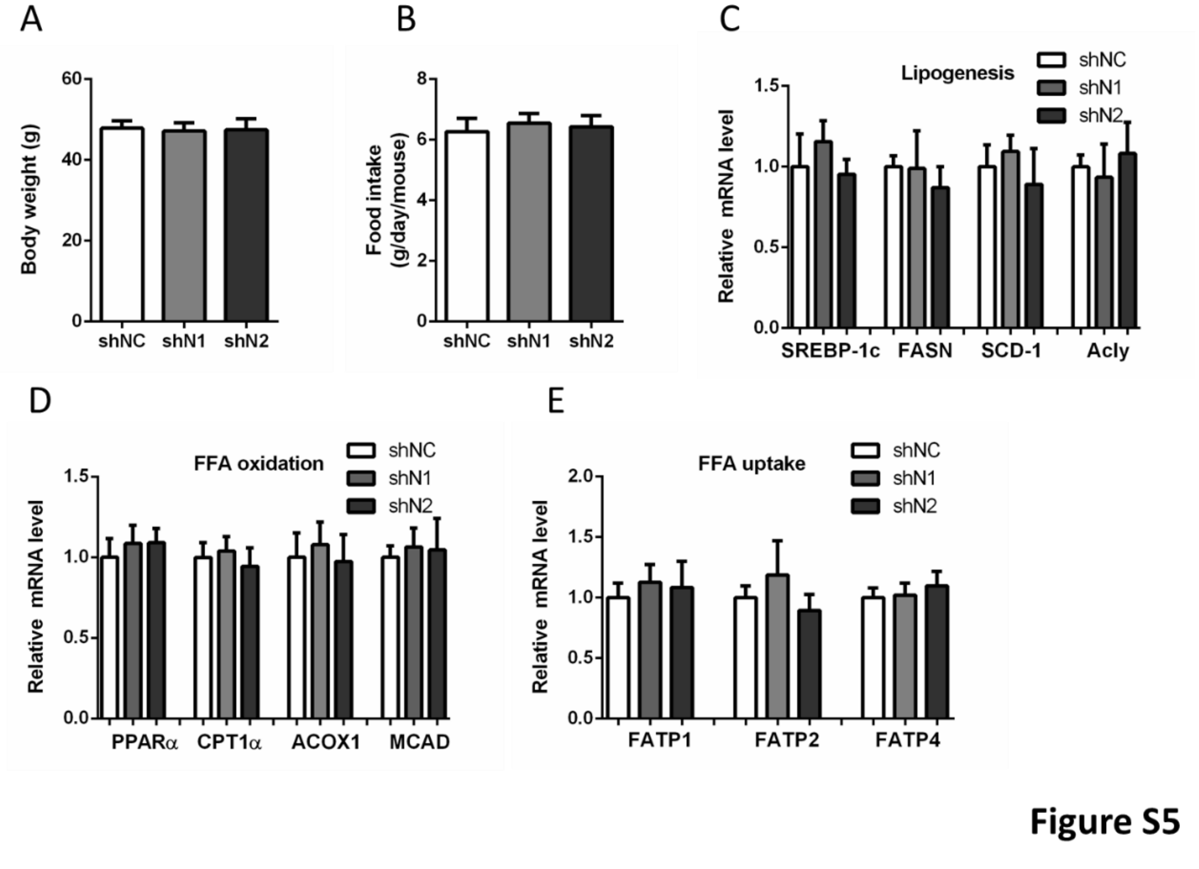

Figure S5. Genes expression in the livers of *db/db* mice infected with adenoviral shRNA. A-B) Body weight and food intake in *db/db* mice administrated with shNC, shN1 or shN2. C-E) Relative mRNA levels of genes related to lipogenesis (C), FFA oxidation (D) and FFA uptake (E) in *db/db* mice. n=6 in each group. Data are presented as mean ± SEM. 1-way ANOVA followed by the

Student-Newman-Keuls test (A-E).

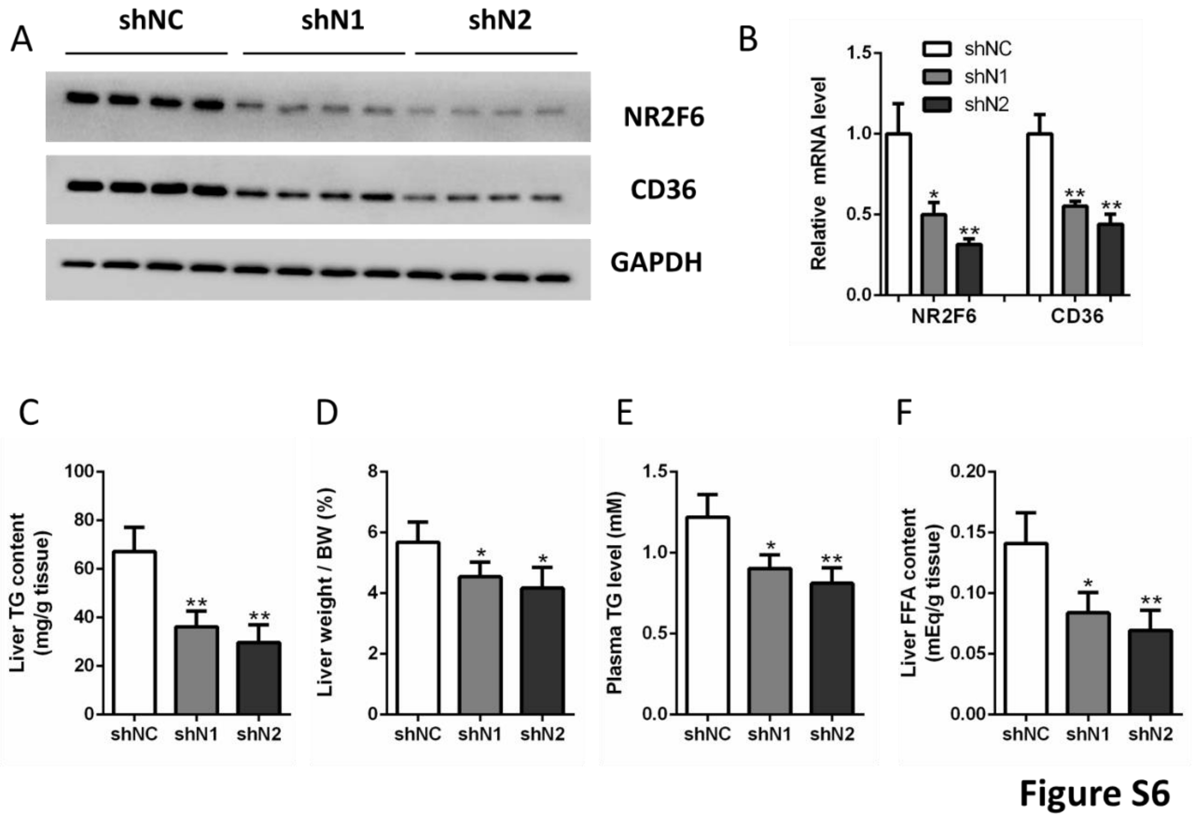

Figure S6. Suppression of NR2F6 improves fatty liver in high-fat-diet-induced obese mice. HFD mice were administrated with two adenoviral NR2F6 shRNAs (shN1, shN2) or negative control (shNC) through tail vein injection and sacrificed at day 14 post-injection. A) Representative protein levels of NR2F6 and CD36 in the livers of HFD mice. B) Relative mRNA levels of NR2F6 and CD36 in the livers of HFD mice. C-F) Liver TG contents (C), liver weight (D), plasma TG levels (E) and hepatic FFA concentrations (F) in the HFD mice. n=6 in each group. Data are presented as mean ± SEM. 1-way ANOVA followed by the Student-Newman-Keuls test (B-F). * $P < 0.05$, ** $P < 0.01$.

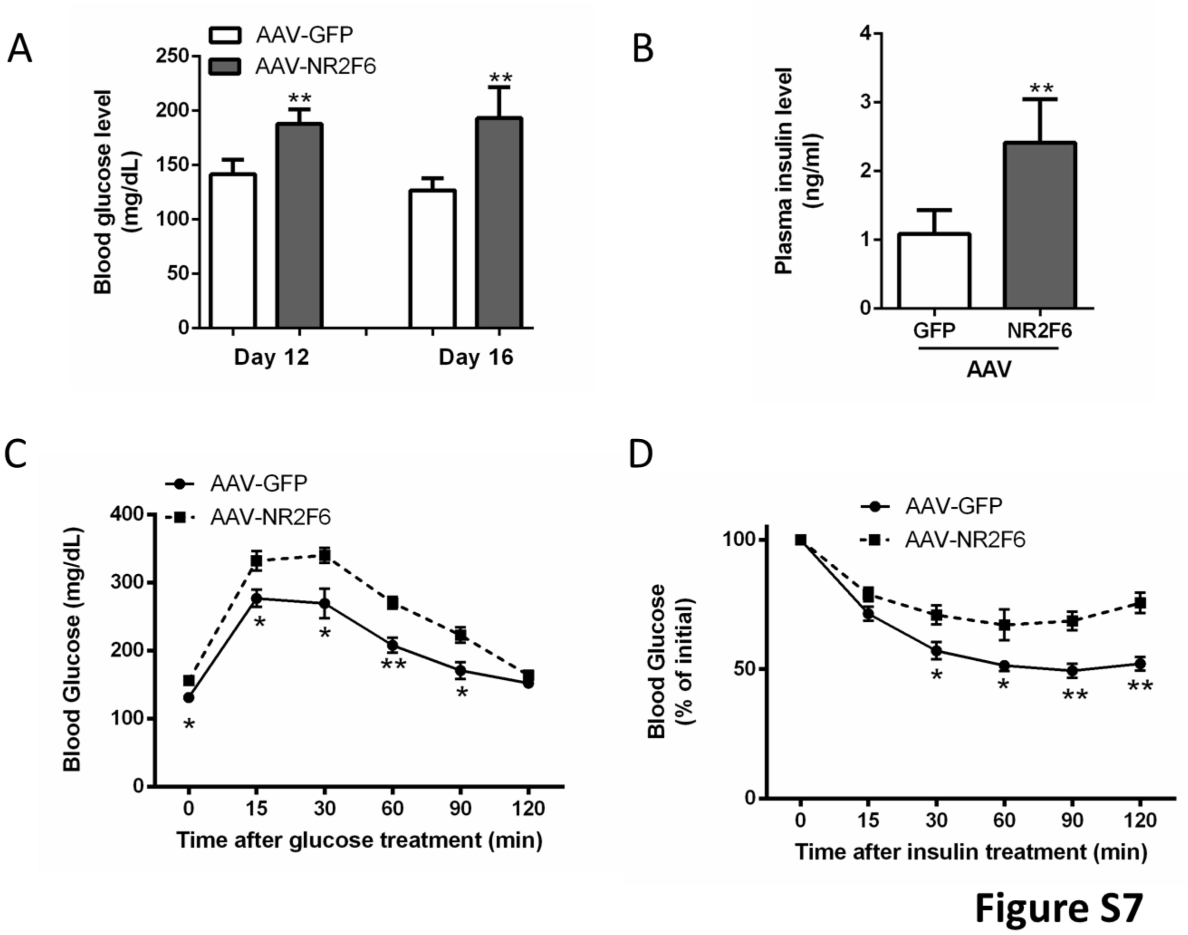

Figure S7. Overexpression of NR2F6 impairs glucose homeostasis. C57BL/6 mice were administrated with adeno-associated virus (AAV9) containing GFP or NR2F6 through tail vein injection and sacrificed at day 30 post-injection. n=5 in each group. A) Blood glucose levels measured after 6 hr of fasting on day 12 and 16 post-injection. B) Plasma insulin levels measured after sacrifice. C) Glucose tolerance test performed on post-injection day 20. D) Insulin tolerance test performed on post-injection day 25. Data are presented as mean ± SEM. 2-tailed Student's t test (A, B). 1-way ANOVA followed by the Student-Newman-Keuls test (C, D). * $P < 0.05$, ** $P < 0.01$.

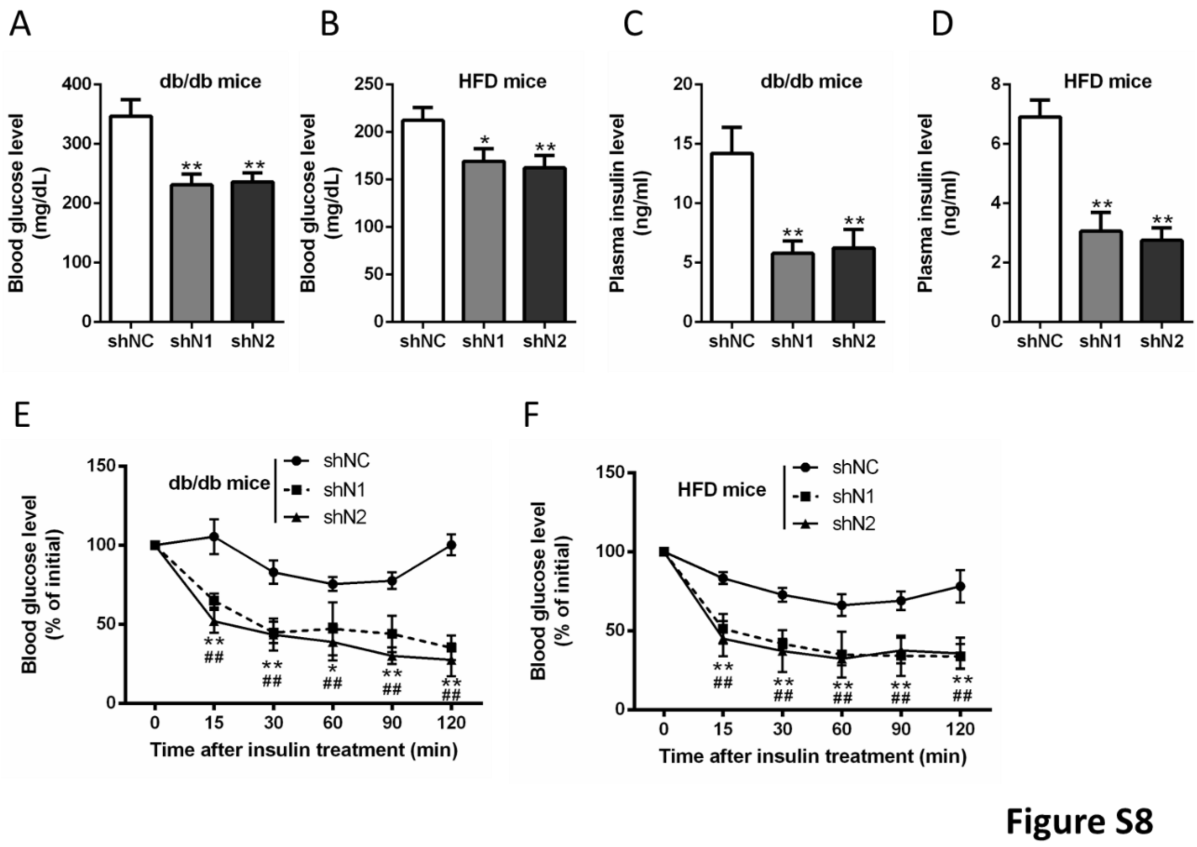

Figure S8. Knockdown of hepatic NR2F6 improves glucose metabolism in obese mice. *Db/db* and HFD mice were administrated with two adenoviral NR2F6 shRNAs (shN1, shN2) or negative control (shNC) through tail vein injection and sacrificed on post-injection day 14. n=6 in each group. A-B) Blood glucose levels measured on post-injection day 6. C-D) Plasma insulin levels measured after sacrifice. E-F) Insulin tolerance test performed on post-injection day 10. Data are presented as mean ± SEM. 1-way ANOVA followed by the Student-Newman-Keuls test (A-F). (E, F) *, ** shN1 versus shNC. ## shN2 versus shNC. * $P < 0.05$, ** $P < 0.01$, ## $P < 0.01$.

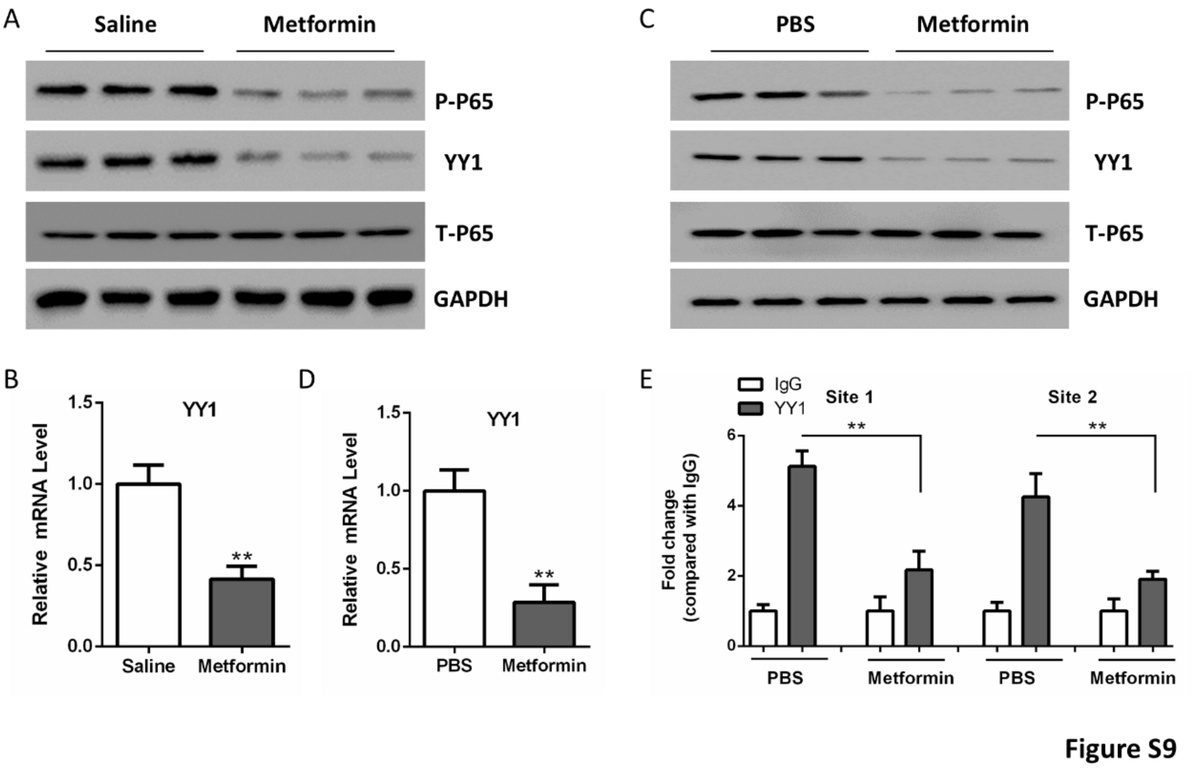

Figure S9. Metformin decreases NR2F6 expression through NF-κB/YY1 pathway. A) Western blots showing the protein levels of phosphorylated P65 and YY1 in the livers of *db/db* mice treated with metformin (50 mg/kg/day) or vehicle control (Saline). B) Relative mRNA levels of YY1 in the livers of *db/db* mice treated with metformin or saline. n=5 per each group. C) Western blots showing the protein levels of phosphorylated P65 and YY1 in the MPHs treated with metformin (0.5mM) or vehicle control (PBS). D) Relative mRNA levels of YY1 in the MPHs treated with metformin or PBS. n=4 per each group. E) ChIP assays showing the binding affinity of YY1 on the two sites of NR2F6 promoter region. n=4 per each group. Data are presented as mean ± SEM. 2-tailed Student's t test (B, D). 1-way ANOVA followed by the Student-Newman-Keuls test (E). ** $P < 0.01$.

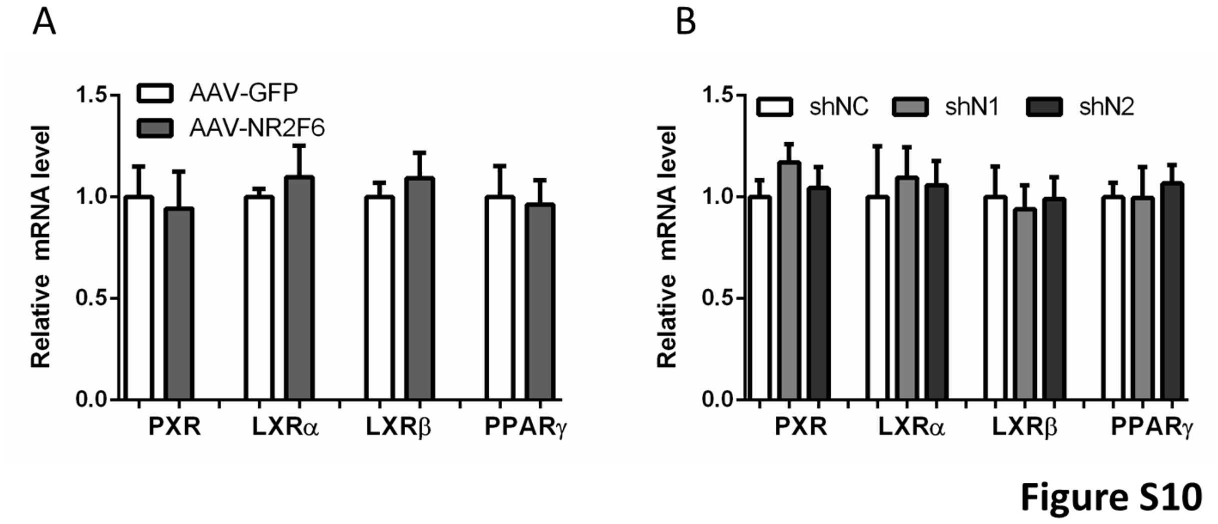

Figure S10. Expression of nuclear receptors in the livers with NR2F6 overexpression or knockdown. A) Relative mRNA levels of nuclear receptors in the livers of C57BL/6 mice expressing AAV-NR2F6 or AAV-GFP. n=5 per each group. B) Relative mRNA levels of nuclear receptors in the livers of *db/db* mice expressing shRNA targeting NR2F6 (shN1, shN2) or control (shNC). n=6 per each group. Data are presented as mean ± SEM. 2-tailed Student's t test (A). 1-way ANOVA followed by the Student-Newman-Keuls test (B).

Table S1: Primers used for quantitative real-time PCR.

Gene	Sequence 5'→3'
Human *NR2F6*	Forward: GAGCGGCAAGCATTACGGT<br>Reverse: GGCAGGTGTAGCTGAGGTT
Human *Rplp0*	Forward: GCAGCATCTACAACCCTGAAG<br>Reverse: CACTGGCAACATTGCGGAC
Mouse *NR2F6*	Forward: GAGGACGATTCGGCGTCAC<br>Reverse: GTAATGCTTTCCACTGGACTTGT
Mouse *CD36*	Forward: ATGGGCTGTGATCGGAACTG<br>Reverse: GTCTTCCCAATAAGCATGTCTCC
Mouse *Rplp0*	Forward: AGATTCGGGATATGCTGTTGGC<br>Reverse: TCGGGTCCTAGACCAGTGTTC
Mouse *SREBP-1c*	Forward: GATGTGCGAACTGGACACAG<br>Reverse: CATAGGGGGCGTCAAACAG
Mouse *FASN*	Forward: GGAGGTGGTGATAGCCGGTAT<br>Reverse: TGGGTAATCCATAGAGCCCAG
Mouse *SCD-1*	Forward: TTCTTGCGATACACTCTGGTGC<br>Reverse: CGGGATTGAATGTTCTTGTCGT
Mouse *Acly*	Forward: CAGCCAAGGCAATTTCAGAGC<br>Reverse: CTCGACGTTTGATTAACTGGTCT
Mouse *FATP1*	Forward: CGCTTTCTGCGTATCGTCTG<br>Reverse: GATGCACGGGATCGTGTCT
Mouse *FATP2*	Forward: TCCTCCAAGATGTGCGGTACT<br>Reverse: TAGGTGAGCGTCTCGTCTCG
Mouse *FATP4*	Forward: ACTGTTCTCCAAGCTAGTGCT<br>Reverse: GATGAAGACCCGGATGAAACG

Gene	Sequence 5'→3'
Mouse *PPARα*	Forward: AGAGCCCCATCTGTCCTCTC<br>Reverse: ACTGGTAGTCTGCAAAACCAAA
Mouse *CPT1α*	Forward: CTCCGCCTGAGCCATGAAG<br>Reverse: CACCAGTGATGATGCCATTCT
Mouse *ACOX1*	Forward: TCCAGACTTCCAACATGAGGA<br>Reverse: CTGGGCGTAGGTGCCAATTA
Mouse *MCAD*	Forward: AGGGTTTAGTTTTGAGTTGACGG<br>Reverse: CCCCGCTTTTGTCATATTCCG
Mouse *MTTP*	Forward: CTCTTGGCAGTGCTTTTTCTCT<br>Reverse: GAGCTTGTATAGCCGCTCATT
Mouse *ApoB*	Forward: AAGCACCTCCGAAAGTACGTG<br>Reverse: CTCCAGCTCTACCTTACAGTTGA
Mouse *ApoC1*	Forward: TCCTGTCCTGATTGTGGTCGT<br>Reverse: CCAAAGTGTTCCCAAACTCCTT
Mouse *ApoE*	Forward: CTGACAGGATGCCTAGCCG<br>Reverse: CGCAGGTAATCCCAGAAGC

Table S1

Table S2: Primers used for plasmids construction.

Plasmid	Sequence 5'→3'
Mouse *NR2F6* Promoter	Forward: GAGCTCGCTAGCCTCGAGAGTCCCCTTATGTGAGGGCTC<br>Reverse: TACCGGATTGCCAAGCTTCTGACACCCGCGTTCCATCGG
Mouse *CD36* Promoter	Forward: GAGCTCGCTAGCCTCGAGGTTAAGTTTAGGTTACACTCATATTC<br>Reverse: TACCGGATTGCCAAGCTTTTCCAGTCCTTCAGACCCACGCC
Mouse *NR2F6* expression plasmid	Forward: GGGAGACCCAAGCTGGCTAGCCGCCACCATGGACTACAAGGACCATGACGGCGAC<br>Reverse: TCCAGCACAGTGGCGGCCGCCTAGCCCGAGCCATAGGGCCAG

Table S2
